# Trial-to-Trial Reoptimization of Motor Behavior Due to Changes in Task Demands Is Limited

**DOI:** 10.1371/journal.pone.0066013

**Published:** 2013-06-11

**Authors:** Jean-Jacques Orban de Xivry

**Affiliations:** ICTEAM and IoNS, Université catholique de Louvain, Louvain-La-Neuve, Belgium; Katholieke Universiteit Leuven, Belgium

## Abstract

Each task requires a specific motor behavior that is tuned to task demands. For instance, writing requires a lot of accuracy while clapping does not. It is known that the brain adjusts the motor behavior to different task demands as predicted by optimal control theory. In this study, the mechanism of this reoptimization process is investigated by varying the accuracy demands of a reaching task. In this task, the width of the reaching target (0.5 or 8 cm) was varied either on a trial-to-trial basis (random schedule) or in blocks (blocked schedule). On some trials, the hand of the subjects was clamped to a rectilinear trajectory that ended 2 cm on the left or right of the target center. The rejection of this perturbation largely varied with target width in the blocked schedule but not in the random schedule. That is, subjects exhibited different motor behavior in the different schedules despite identical accuracy demands. Therefore, while reoptimization has been considered immediate and automatic, the differences in motor behavior observed across schedules suggest that the reoptimization of the motor behavior is neither happening on a trial-by-trial basis nor obligatory. The absence of trial-to-trial mechanisms, the inability of the brain to adapt to two conflicting task demands and the existence of a switching cost are discussed as possible sources of the non-optimality of motor behavior during the random schedule.

## Introduction

Playing bowling requires a lot of flexibility in motor behavior. Both the choice of a heavy or light ball and the number of skittles remaining at the end of the bowling lane influence the ball throw. For instance, if there is only one skittle remaining at the end of the bowling lane, the accuracy of the throw should be prevalent while the presence of multiple skittles should typically decrease its importance for any naïve player. The motor behavior used when a single skittle is present could be used when many of them are still standing. A player could focus on one of the many skittles (e.g. the middle one) and throw with the same motor behavior as in the one-skittle situation. However, the brain appears to adjust the control policy in order to take the task demands into account [1], [2]. Varying task demands (e.g. the number of skittles) can thus shed light on the updating mechanisms of the motor control policy.

Optimal control 3–7 provides a good theoretical background to understand the influence of change in the dynamics of the world [8]–[12] or of accuracy demands on motor behavior [1], [2]. In this framework, the control policy is shaped by a cost function that includes accuracy demands, energy expenditure, reward, etc. This control policy determines both the kinematics of the motor behavior but also how perturbation will be rejected. Following this theory, a movement will be deemed optimal if it minimizes energy expenditure while maximizing reward and accuracy. Modifying any of the components of the cost function influences the control policy accordingly, hence the kinematics of the movement and how a perturbation is rejected. For instance, varying the amount of energy that needs to be spend to achieve a goal influences interlimb coordination [13]. Similarly, an increase in reward rate or more rewarding stimuli make the eyes move faster during a saccade [14]–. Finally, accuracy demands modulate feedback gains within the control policy on a trial-by-trial basis [1], [2], [11]. These studies concluded that the influence of accuracy demands on motor behavior is obligatory and that the motor behavior is always optimal with respect to a given cost function.

In the present study, the notion of obligatory reoptimization of the motor behavior is challenged. This reoptimization was elicited by varying the width of the target that the subjects were instructed to reach to. The motor behavior was studied in a context where accuracy demand changes from trial-to-trial and a context where it remains constant for several trials in a row. In the optimal control framework described above, the cost function varies with target width independently of the context and these contexts should not influence the motor behavior. However, the comparison of the motor behaviors observed in these two contexts revealed differences that shed new lights on the mechanisms that govern the reoptimization of the motor behavior.

## Methods

### Subjects

Fifty-one healthy subjects (20 for experiment 1, 10 for experiment 2, 12 for experiment 3 and 9 for experiment 4) were enrolled for the experiments after written informed consent. All subjects had no history of neurological disorders, were right-handed and between 18 to 40 years old. All procedures were approved by the Ethics Committee of the Université catholique de Louvain.

### Setup

Subjects were sitting in front of a robotic arm. They controlled the handle of the robot in order to move a cursor that was displayed on a horizontal mirror positioned above the arm. The cursor and targets of interest were displayed on a screen placed tangentially above the mirror and were reflected by it. Because the mirror was halfway between the handle and the screen, the cursor appeared to be positioned at the same position in space as the hand once the device was properly calibrated. With this setup, subjects could not see their hand and the displayed cursor was the only available visual feedback of their arm position.

The robot (Endpoint Kinarm, BKin Technologies, Kingston, Ontario, Canada) monitored hand position, velocity and acceleration at 100 Hz. In addition, a force transducer monitored the force exerted by the subjects on the handle of the robot. Kinematic and dynamic data were stored on a PC for offline analysis.

The robot was also able to exert forces in order to perturb or direct the hand of the subjects. The robot was controlled by a real-time computer running custom-made programs written in Matlab with the help of the StateFlow toolbox. The same program controlled the display of the stimuli on the screen.

### Protocol

Four different experiments were conducted to test the influence of target width on motor behavior. In experiments 1 and 2, subjects were instructed to reach to targets with varying widths. These experiments differ in the number of trials received in the random and blocked schedules (see below). The number of trials in each of the schedule was similar for experiments 2, 3 and 4 but experiment 3 and 4 involved shooting movements rather than reaching movements. In experiment 4 but not in experiment 3, the width of the target on the next trial was presented well in advance of the time of target presentation.

For all experiments, each trial started with the appearance of a 25 mm^2^ red square (the starting position) that was located in the middle of the screen, 15 cm ahead of the subject. The robot pulled the hand of the subjects in order to bring it inside the square. As soon as the hand cursor was stabilized inside the starting point, the square became green and a variable delay elapsed (500–600 ms for experiments 1–3, 700–800 ms for experiment 4) before a target appeared 15 cm away from the starting position (30 cm in front of the subject). The subjects were instructed to move the cursor inside the target within a time interval of 500 to 600 ms. When the subjects stopped on the target, the cursor turned blue, green or yellow depending on movement time (blue = too slow, yellow = too fast, green = good speed). When movement time was within the instructed range, the subjects earned one point that was added to the total number of points collected so far and displayed after each movement. Another point could be earned with the same movement based on an accuracy criterion (within the target at movement end). Movement onset and offset were detected online with position and velocity criteria. Movement onset was flagged when the hand left the area of the starting position and hand velocity was higher than 0.02 m/s. Movement end was detected when hand velocity was lower than 0.02 m/s and hand position less than 1 cm away from the target along the dimension parallel to the movement for experiments 1 and 2 and when the distance travelled was larger than 15 cm for experiments 3 and 4. This endpoint error measure does not constrain the variability of movement endpoint along the lateral dimension. If the hand reached velocities higher than the velocity threshold after the detected movement end, movement time was incremented and target color was updated accordingly. A few hundred of milliseconds after movement end, the hand was pushed back towards the starting position while the hand cursor was removed from the screen.

The experiment was divided into blocks of trials that were separated by a one-minute break. All subjects started the experiment with a practice block. During this block, they performed 60 movements towards a 25 mm^2^ square target with a small hand cursor (cursor was 9 mm^2^) that provided online visual feedback of hand position to the subjects. This practice block allowed subjects to conform to the speed requirement of the task. The data from this block was not collected for a subset of the subjects and was not analyzed further.

**Figure 1:**
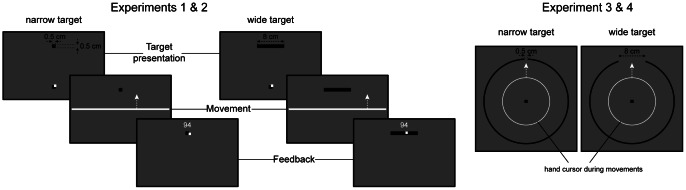
Protocol. For each trial, the target was presented 15 cm away from the starting position in front of the subject (target presentation phase). The width of the target could either be 0.5 cm, or 8 cm while its length stayed constant (0.5 cm). As soon as the hand of the subject left the starting position (movement phase), the cursor indicating the position of the hand with respect to the target became a very large horizontal bar that spanned the width of the screen. Veridical hand position was provided after movement end in order to provide feedback about movement accuracy (feedback phase). The target became blue if movement duration was too long, yellow if movement duration was too short and green if movement duration was between 500 and 600 ms. One point could be earned for good movement speed and another point could be earned for good accuracy. The total number of points collected so far in the block was displayed at the end of each trial (94 points in the illustration). For experiments 3 and 4, subjects were instructed to pass through the opening in a circle rather than to stop on a rectangular target (right panel). The hand cursor was replaced by a circle whose radius increased with the distance travelled by the hand.

After the practice block and for the rest of the experiment (4 experimental blocks for experiment 1 and 3 experimental blocks for experiments 2, 3 and 4), a modified hand cursor was used during the movements towards the target (Movement phase, left panel of Fig. 1). This cursor was 80 cm long and 3 mm wide (experiments 1 and 2). It did not yield any feedback about the lateral position of the hand but yielded veridical information about the distance travelled by the hand towards the target. Outside the movement period, the hand cursor was a 9 mm^2^ square. Therefore, as soon as the movement ended, the cursor width was reduced, which allowed the subjects to perceive their endpoint error.

In some trials (perturbation trials), the hand path was constrained by stiff virtual walls. These walls were created by applying a stiff uni-dimensional spring (spring stiffness: 2500 N/m and viscosity: 25 Ns/m) towards the target or 2 cm either on the left or the right of it. The force exerted by the subject to oppose the perturbation was measured by the force transducer and used as a proxy of how the control policy rejects any perturbation to the movement.

**Figure 2:**
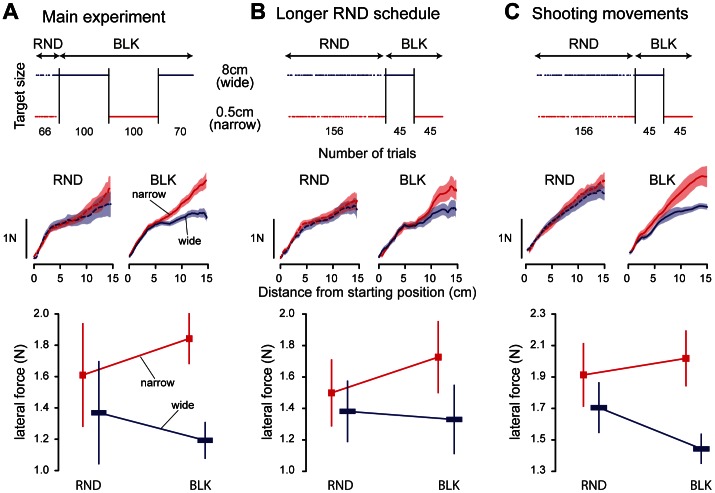
Forces exerted against the perturbation in the three experiments. Top row describes the distribution of target width over the course of trials. In the first experiment (panel A), target width was randomly assigned for the first 66 trials while it was randomly assigned for the first 156 trials in the two other experiments. In the blocked schedules, target width changed every 100 trials in the first experiment with a maximum of 270 trials. In the second (panel B) and third experiments (panel C), target width changed only once after 45 trials. Middle row depicts the average force profiles across all subjects recorded during the perturbation trials for each schedule and each target width separately. In these plots, the forces are represented against the distance from the starting position in order to match the level of perturbation. Shaded area around each curve represents standard error of the mean. The bottom row presents the statistical analysis of the forces recorded at 13 cm in the force profiles in function of target width and schedule. Each column represents a different experiment. Error bars represent the 95% confidence interval.

For the main experiment (top row of Fig. 2A), the width of the target varied randomly from trial to trial (0.5 or 8 cm; random schedule) during the first experimental block (66 trials), but target length was maintained constant (0.5 cm). Target width only was modulated in order to avoid any effect of the speed-accuracy trade-off [18]. Sixteen perturbation trials were randomly interspersed during the last 56 trials of the block (i.e. the first ten trials were unperturbed). The next three experimental blocks lasted 90 trials each. In these blocks, the target width was maintained constant for 100 consecutive trials (blocked schedule). Perturbation trials were also pseudo-randomly interspersed (10 left perturbations, 10 right perturbations and 5 straight ahead perturbations). In the WNW group (Wide-Narrow-Wide, ten subjects), the wider target was presented first whereas in the NWN group (Narrow-Wide-Narrow, ten subjects), the narrower target was presented first.

In experiment 1, there were fewer trials in the random schedule than in the blocked schedule. This difference might affect the influence of schedules on motor behavior (see Results). To control for the number of trial, the number of trials in the random and blocked schedules was, respectively, increased and decreased in a second experiment. In this second experiment, a second block of 90 trials in the random schedule was presented after the first experimental block (top row of Fig. 2B). The third experimental block contained 45 trials with the wide target followed by 45 trials with the narrow target (blocked schedule). In these series of 45 trials, 5 left perturbations, 5 right perturbations and 3 straight ahead perturbations were pseudo-randomly interspersed.

In the first two experiments, the modulation of the force against the perturbation was especially pronounced at the end of the movement (see results). To test whether the reported effect was related to the increased stiffness observed at the end of movements [19], [20], reaching movements were replaced by shooting movements. In the third experiment (right panel of Fig. 1), eleven subjects were instructed to shoot through the targets and not to stop on it. For this experiment, target presentation was modified. At the start of each trial, a circle (diameter of 15 cm) was presented on the screen and centered on the starting position. At the location of the targets of experiments 1 and 2, the circle was open. This opening had a width of either 0.5 or 8 cm. The subjects were instructed to exit the circle through the opening. During the movement, the hand cursor was replaced by a circle that was centered on the starting position and that had a radius that corresponded to the distance travelled by the hand. All other aspects of the experiment remained similar to experiment 2. One subject was excluded from the analysis because of a sudden 3-fold increase in force during the random schedule for both target widths.

In the blocked schedule, target width can be predicted well before movement onset while target width only becomes available at the time of target presentation in the random schedule. The aim of the fourth experiment was to test whether the predictability of target width could explain the observed differences between the random and blocked schedules. Experiment 4 was identical to experiment 3 except that 1) a cue was presented immediately before the starting position and 2) the delay between reaching the starting position and target appearance was raised to 700–800 ms. The cue indicated the width of the target on the next trial in order to render target width predictable. The cue for the narrow (resp. wide) target was a 0.5 by 0.5 cm (resp. 8 by 0.5 cm) orange square (resp. rectangle) displayed 3 mm below the starting position. The cue disappeared after reach onset.

### Data Analysis

In perturbation trials, we used the lateral force exerted by the subjects against the wall of the channel as a proxy of their willingness to go straight towards the target. Force measures were low-pass filtered (second order Butterworth filter with cutoff: 75 Hz). The lateral force from straight ahead perturbation trials was subtracted from the measures of lateral force during rightward or leftward perturbation trials separately for each schedule and target width separately. These corrected measures were sign reversed for the rightward perturbations such that a larger positive force represents a larger reaction to the perturbation. To quantify the reaction to the perturbation, we extracted the lateral force exerted by the subjects when they were 2 cm away from the target. These 2 cm allowed us to avoid late correction of the movements or the period where the cursor was back to normal size.

In unperturbed trials, our proxy for movement performance was the position of the hand when it was 2 cm away from the target in depth. This measure was considered as movement endpoint and both its mean and standard deviation were used. Unperturbed trials were excluded from these analyses if they immediately followed a perturbed trial. To assess the trial-to-trial changes in movement endpoint, the auto-correlation with lag 1 was computed [21], [22]. Theoretically, the planned endpoint of the next movement (*m^k+1^*) is updated by adding some fraction (*B*) of the error during the previous movement (*err^k^*) to the planned endpoint of the previous movement (*m^k^*). Therefore, this relationship, which is corrupted by noise, can be written as [21], [22]:
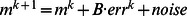



If the error is not taken into account (*B* = 0), the correlation between the endpoint of two consecutive movements should be high because only noise disrupts the relationship between those two variables. In contrast, this correlation should be zero or negative [23] if the error is taken into account (*B* = 1 and *err^k = ^T-m^k^*). For each subject separately, each pair of consecutive trials that did not include a perturbation trial and were in the same schedule was included in this analysis. The auto-correlation was performed separately for each target width in the blocked schedule but not in the random schedule.

ANOVA was used with schedule (random or blocked) and target width (narrow and wide) as within-subjects factors. Tukey post-hoc test was used to assess the significance of pair-wise comparisons. Significance level is 0.05. For illustration purposes (Fig. 2), force profiles are represented versus distance travelled from 300 ms before to 300 ms after the time of peak velocity.

## Results

To address the question of the influence of accuracy demands on motor behavior, we asked subjects to reach to a target that had a variable width (Fig. 1). In the first 66 trials, the target width changed randomly (top row of Fig. 2A; random schedule - RND) while it stayed constant for a series of at least 70 movements for the rest of the experiment (blocked schedule - BLK).

A typical measure of the effect of target width on kinematics is the variability of movement endpoint. As found in many other studies, the variability of the movement endpoint was larger for the wide target than for the narrow target (main effect of target width: F(1,18) = 17.12, p = 0.0006). This variability was reduced in the blocked schedule (main effect of schedule: F(1,18) = 9.16, p = 0.007). There was no evidence of an influence of the schedule on the variability of movement endpoint (interaction between schedule and target width: F(1,18) = 1.79, p = 0.2). Importantly, this is independent of the speed-accuracy trade-off as the change in accuracy was orthogonal to the movement speed.

To probe the control policy of the motor plant further, perturbation trials were interspersed. During these trials, the hand was directed to the left and to the right from the center of the target in a channel and the force that the subjects exerted against the perturbation was measured. This perturbation did not affect the visual information provided to the subjects. The average force profiles for the random and blocked schedules are displayed in the middle row of Fig. 2.A. The force exerted by the subjects increased with distance because of the increasing magnitude of the perturbation. The force exerted by the subjects to counteract the perturbation was only slightly modulated by target width in the random schedule (left panel). In contrast, in the blocked schedule, the force was larger for the narrow target than for the wide target (right panel).

To summarize this observation we computed the force at 13 cm in order to remove any effects of stopping at the target. We did not find a significant effect of schedule but a main effect of target width and an interaction between condition and target width (bottom row of fig. 2.A; interaction between schedules and target width: F(1,18) = 16.25, p = 0.0008). This interaction suggests that the difference in force between the two targets was larger for the blocked (narrow vs. wide: post-hoc test: p = 0.0002) than for the random schedule (narrow vs. wide: post-hoc test: p = 0.02). The change in behavior could consist either in a decrease in force between the two schedules for the wide target or to an increase in force for the narrow target. Between the two schedules, 80% of the subjects exhibited an increase in force for the narrow target while 60% of the subjects decreased their force for the wide target.

The weak modulation of motor behavior with accuracy demands during the random schedule might stem from an absence of trial-to-trial adaptation of the control policy. Such a trial-to-trial mechanism would imply that the width of the target experienced on one trial would affect the behavior on the next trial. That is, the force applied to counteract the perturbation would be larger when the perturbation trial was preceded by a trial with a narrow target than when it was preceded by a trial with a wide target. This effect should be independent of the width of the target presented for the perturbation trial. Therefore, the influence of the target width of the trials that preceded a perturbation trial on the response to the perturbation was investigated. The width of the target on the previous trial did not modulate the force exerted by the subjects against the perturbation in the next trial. That is, the force was not larger when a trial was preceded by a narrow target than when it was preceded by a wide target (main effect of previous trial; p = 0.77). This observation suggests that there was no evidence of a trial-to-trial adaptation of the behavior.

The smaller effect of target width on force in the random schedule might be due to the short length of the random schedule (66 trials). To test this hypothesis, a second experiment was conducted with ten subjects where the length of the random schedule was 2.5 times longer and the blocked schedule was three times shorter than in the initial experiment (top row of Fig. 2B). Increasing the number of trials in the random schedule did not lead to an increase in force modulation with target width (middle row of Fig. 2B). The inter-subject average force profiles were influenced by target width in the blocked schedule, similarly to what was observed in the experiment 1. The difference was still larger in the blocked than in the random schedule (interaction between target width and schedule: F(1,9) = 5.9, p = 0.038; post-hoc tests: RND: narrow vs. wide: p = 0.5; BLK: narrow vs. wide: p = 0.004). The influence of target width from previous trial on the force exerted in the next trial failed to reach significance despite the increased number of perturbation trials in the random schedule (F(1,9) = 3.57, p = 0.09). Therefore, even with more trials in the random schedule, there was no evidence for a trial-to-trial adaptation of the motor behavior.

The increase in stability requirement at the end of reaching movements [20] could drive the observed effect. To test this hypothesis, a new experiment was conducted during which eleven subjects were instructed to shoot through the target rather than to stop on the target (right panel of Fig. 1). The length of the random and blocked schedule was similar to experiment 2 (top row of Fig. 2C). Again, the force profiles indicated that target width did not influence the reaction to the perturbation in the random schedule but did modulate it in the blocked schedule (middle row of Fig. 2C). The influence of target width on force during perturbed trials was, for the third time, larger in the blocked than in the random schedule (interaction between target width and schedule: F(1,10) = 10.64, p = 0.009; post-hoc tests: RND: narrow vs. wide: p = 0.1; BLK: narrow vs. wide: p = 0.003). In the random schedule, trial-to-trial adaptation of the motor behavior was again absent. The target width experience on one trial did not influence the force exerted against the perturbation on the next trial (F(1,10) = 0.21, p = 0.65).

Finally, a fourth experiment was performed in order to test the effect of target width predictability on the reoptimization of the control policy. Indeed, target width for the upcoming movement was unpredictable in the random schedule but predictable in the blocked schedule. This unpredictability of upcoming target width could explain the absence of reoptimization of the control policy in the random schedule. This fourth experiment was identical to experiment 3 except that a cue was displayed below the starting position at the start of each trial (around 1 s before movement onset). The cue non-ambiguously indicated the width of the target on the upcoming trial. Despite this additional information, the modulation of the force with target width was still larger in the blocked schedule than in the random schedule (interaction between schedule and target width: F(1,8) = 8.7, p = 0.018). There was no sign of trial-to-trial adaptation of the control policy (main effect of the target width on the previous trial: F(1,8) = 3.74, p = 0.09). It indicates that the absence of information about future target width cannot explain the difference in motor behavior between the random and blocked schedules.

**Figure 3:**
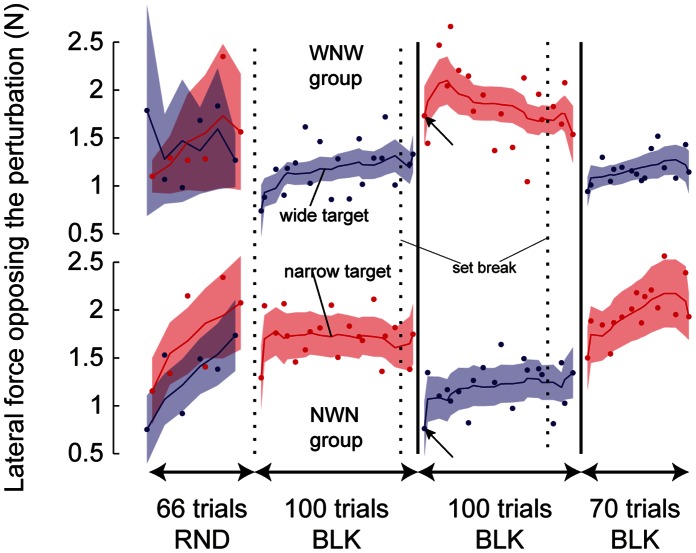
Evolution of the lateral force opposing the perturbation over the course of trials during the first experiment. The two groups of subjects, which differ in the target width received first during the blocked schedule, are represented in the top and bottom rows. Dotted black vertical lines represent set breaks while solid black vertical lines mark a change in target width during the blocked schedule. Black arrows highlight the force exerted during the first perturbation trials after a change in target width in the blocked schedule. RND: random schedule; BLK: blocked schedule.

The absence of trial-to-trial adaptation of motor behavior observed in each experiment is reinforced by the observation that, in the blocked schedule, the evolution of the force over the course of trials does not follow a gradual trial-by-trial change. Indeed, the transition from one target width to the other one did not follow an exponential change such as during motor learning but reflected a rather abrupt transition. Fig. 3 represents the change in force over the course of the trials for the two groups of subjects from experiment 1. In the blocked schedule, the force very quickly reached a plateau level. There does not seem to be a gradual change of its force. Rather, the change in force occurred very quickly (black arrows in Fig. 3, first perturbation trial was trial #5 after the change in target width). A gradual change in force would result in the first perturbation trial after the change in target width being larger if the subjects first experienced a narrow target (narrow-wide-narrow (NWN) group, bottom row of Fig. 3) than if they experienced the wide target first (wide-narrow-wide (WNW) group, top row). The first perturbation trial after the change in target width was different in both groups. Subjects that then received the narrow target after many trials with the wide target exerted a larger force than the subjects that received the wide target after a long series of trials with the narrow target (t(18) = 2.3, p = 0.033). That is, the current target width determined the amount of force, not the long history of trials before then.

The order of the target width in the blocked schedule did not modulate the amount force exerted by the subjects. Namely, the history of target presentation did not influence the motor behavior (interaction between order of presentation and target width: F(1,18) = 0.89, p = 0.36). This analysis suggests that the force exerted by the subjects was identical when they were first presented with the narrow target than when they were presented with the wide target first.

**Figure 4:**
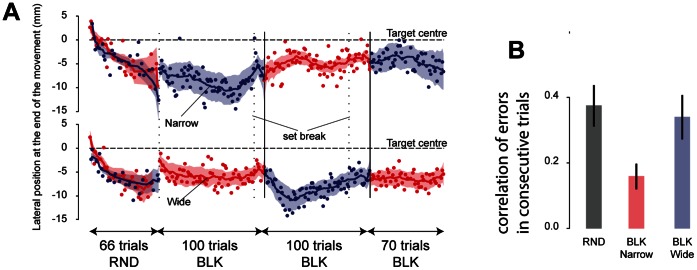
Movement endpoints. **A**) Evolution of the lateral position at the end of the movement over the course of trials during the first experiment. The two groups of subjects, which differ in the target width received first during the blocked schedule, are represented in the top and bottom rows. Dotted black vertical lines represent set breaks while solid black vertical lines mark a change in target width during the blocked schedule. Dashed horizontal line represents the lateral position of target center. RND: random schedule; BLK: blocked schedule. **B**) Autocorrelation of lag 1 of movement endpoint for the random schedule and each target width in the blocked schedule. A large positive correlation indicates low trial-to-trial error correction while a low or negative correlation indicates a high trial-to-trial error correction.

In unperturbed trials, the position of the hand was recorded immediately before the subjects reached the target (at 13 cm). The lateral position was measured with respect to the middle of the target. A slight anisotropy of the system made the subjects reached slightly to the left of the center of the target (Fig. 4A). Throughout the experiment, the reaching endpoints were biased towards the left of the target center. This bias was modulated by the schedule and by the target width. It varied similarly for the narrow and wide targets during the random schedule. It remained small for the narrow target in the blocked schedule but was much more variable when the target was wide in the blocked schedule. The average value of the offset depended on the interaction between target width and schedule (F(1,18) = 15.04, p = 0.001).

While an external change in accuracy demands does not influence the control policy on a trial-to-trial basis, the absence of large offset for the narrow target in the blocked condition suggests that a trial-to-trial error correction mechanism keeps movements accurate by updating the aimed direction [21]. Such a mechanism should reduce the correlation between the endpoint positions of consecutive movements [see Methods and 22]. In the blocked schedule, this correlation was higher for the wide target than for the narrow target (Fig. 4B, t(38) = −2.47, p = 0.015). This difference in autocorrelation indicates that, in the blocked schedule, movement endpoint was more actively controlled when the target was narrow than when it was wide. The control of movement endpoint was also limited in the random schedule. Indeed, the autocorrelation during the random schedule (narrow and wide targets together) was higher than the autocorrelation for the narrow target during the blocked schedule (t(38) = −3.1, p = 0.004). The autocorrelation during the random schedule did not differ from the autocorrelation for the wide target of the blocked schedule (t(38) = −0.4, p = 0.69). This high autocorrelation of movement error indicates that, during the random schedule, the error observed in one trial is not well taken into account in order to update the aimed direction on the next trial.

Despite the absence of trial-by-trial error correction mechanisms for the wide target in the blocked schedule, there was a reduction of the error in some cases (Fig. 4). This error reduction was quantified by computing the difference between the maximum offset and the value of the offset by the end of the block (average over last five trials) for each subject separately. We found that this reduction of the offset was quite large (7±5 mm, mean±SD) and significantly different than zero (t(19) = −6.62, p<0.0001). This error reduction is not linked to trial-to-trial mechanisms because these are absent for the wide target during the block schedule (see above) and therefore points to a strategic correction of the planned movement direction, which might be similar to strategic correction found in motor learning tasks [24].

## Discussion

In the present study, task demands was varied in order to elicit a reoptimization of the control policy as predicted by optimal motor control theory [1]–[3]. Different motor behaviors were observed between different schedules despite identical task demands. That is, the influence of accuracy demands on the control policy was much larger when accuracy demand was kept constant for several trials in a row (blocked schedule) than when it varied on a trial-by-trial basis (random schedule). This result suggests that the control policy is not optimal in the random schedule. It contrasts with previous studies that suggested that reoptimization was obligatory and restricted to feedback gains [1], [2]. In the present study, the visual feedback was unavailable during the movement in order to diminish the influence of online control mechanisms based on visual feedback and to increase uncertainty about the state of the arm. This manipulation does not abolish the online control of movement [25]. However, optimal control theory [4] suggests that removing visual information should increase the uncertainty about the state of the limb, which should in turn result in a reduction of the feedback gains. Therefore, making sensory information less reliable might uncover new features of the reoptimization process.

### What is Changed in the Behavior?

The control policy that was measured in the perturbation trials was clearly affected by a change in task demands. Following the minimum intervention principle [3], reaction to the perturbation was larger when the target was narrow than when it was wide. Movement endpoint variability was also larger for the wide than for the narrow target and the update of the planned aiming direction varied with accuracy demands and target schedule (Fig. 4, [21], [22]).

Several mechanisms have been proposed to account for changes in control policy with task demands [11]. For instance, limb impedance is modulated by accuracy requirements in a blocked schedule [26], [27] and co-contraction during movement vary with target width in a random schedule [20]. Note that limb impedance or co-contraction at movement endpoint can be modulated on a trial-by-trial basis to reflect change in stability demands [19], [20]. However, the shooting experiment (#3) suggests that endpoint stability is not a major factor in the present study. Long-latency feedback gains are also modulated by target width both in the random and blocked schedules [1] and so are visual feedback gains in the random schedule [2]. Such feedback gains are sensitive to task-relevance [28].

It is hard to reconcile these findings with the current results because very few studies found differences between the random and blocked schedules [1], [19]. In addition, modulation of limb impedance and of feedback gains have been shown to vary on a trial-to-trial basis [1], [20]. Changes in the control policy cannot be assigned to one mechanism (e.g. modulation of feedback gains) in the random schedule and the other mechanism (e.g. modulation of limb impedance) in the blocked schedule.

Alternatively, different components of the movement could be reoptimized in the two schedules. Each movement can be decomposed as a feedforward, open-loop component and a feedback component and can be modeled this way by optimal control theory [29]. For instance, Knill and colleagues [2] considered that the feedforward component was unaffected by accuracy demands and that movement flexibility was determined by the adjustment of the feedback gains only [1], [2]. This trial-to-trial adjustment of the feedback controller has also been found in force-field adaptation tasks [30], [31]. The reoptimization of the control policy might be restricted to the feedback gains in the random schedule but not in the blocked schedule where the feedforward component could be reoptimized as well. The reoptimization of one or both components of the movement might explain why a change in accuracy demands had a larger impact in the blocked schedule compared to the random schedule. This hypothesis warrants further investigation.

### Reoptimization Due to a Change in Task Demands is not Gradual

In a changing world, animals or humans can either switch behavior when necessary [32]–[34] or gradually adapt their behavior on the basis of performance [35], [36] or reward outcome through Hebbian learning [37]–[40]. In the present experiment, the reoptimization of the control policy elicited by a change in accuracy demands did not conform to a trial-by-trial mechanism. Indeed, there was no effect of the target width during one trial on the response to the perturbation on the next trial and there was not after-effect after a long series of trials with the same target width (Fig. 3). These observations suggest that either trial-to-trial mechanisms are perturbed in the random schedule or the reoptimization process does not conform to a trial-to-trial mechanism.

### Reoptimization Due to a Change in Task Demands is not Obligatory

Absence of reoptimization of the control policy has been observed in experiments where the control policy had to take into account changes in motor uncertainty [41] and where muscle coordination was modified but the updated control policy did not conform to optimality [12]. The latter results were interpreted as evidence of absence of optimality of motor behaviors and the presence of habitual behavior that could hardly be modified. In this study, the absence of reoptimization could not be due to an impossibility to compute the optimal solution because this optimum was observed in the blocked schedule. Therefore, the present results suggest that the reoptimization is not obligatory even when it is possible (i.e. in the random schedule) but takes place in situations where the environment is sufficiently stable, namely in the blocked schedule. In this respect, the process of reoptimization contrasts with error-dependent processes that are obligatory and without conscious awareness [42], [43]. This absence of reoptimization with a change in task demands in the random schedule could be due to one of three reasons. First, trial-to-trial mechanisms are abolished during the random schedule. In this case, if reoptimization occurs on trial-to-trial basis, reoptimization is also blocked. Second, it is not possible to optimize a single control policy to two different task demands concurrently. Third, reoptimizing the behavior to account for a change in task demands bears some cost (switching cost).

The suppression of all trial-to-trial mechanisms during the random schedule would be consistent with two observations. First, the width of the target size on one trial does not influence the response to the perturbation on the next trial. Second, errors are less taken into account in the random schedule than in the blocked schedule (Fig. 4). However, there are numerous examples that demonstrate that the brain learns from past experience even in an uncertain world [23], [44]. Sometimes, the learning is even greater when uncertainty is larger [45].

The inability of the brain to optimize a single control policy for two different task demands can be compared to the inability of the brain to adapt to two different sensorimotor transformations concurrently. Learning two different sensorimotor transformations is very limited when the sensory input is identical [46]–[48]. However, when two different tasks that require two different control policies are used, the feedback control policy associated with each of the task can be adapted independently from the other [31], [49]. In this case, each of these feedback controllers can be adapted to a sensorimotor transformation that is in conflict with the other. In the present study, it is likely that a single control policy is used for the narrow and wide targets. Our results suggest that updating this single control policy for each trial would not be possible. Alternatively, the control policy could be optimized over several trials and not on a single trial basis as suggested for the bouncing ball task [50]. In this case, the cost function would be influence by the schedule despite identical task demands and movements would be deemed optimal in both schedules.

Finally, the idea of a switching cost is also present in cognitive tasks such as symbol classification. In a symbol classification task, subjects are presented with a character pair that contained a digit and a letter that they have to classify as odd or even or as consonant or vowel [51]. To indicate their choice, they had to press one of two keys from a computer keyboard and the reaction time is measured as a probe of movement preparation. The goal of the task changed predictably every two trials. Despite the predictability of the task switch, it takes longer for a subject to press a key in order to indicate whether the digit was odd or even if the task on the previous trial was letter classification than if it was digit classification. This shows that switching the control policy because of a change in task demands carries some cost, as demonstrated by the increase in reaction time [52]. In this study, the small influence of accuracy demands on the behavior in the random schedule might be due to the cost of switching the control policy. Therefore, reoptimization of the motor behavior is not obligatory but is weighted against the cost of switching the behavior. Habitual motor behavior is preferred in the random schedule because it costs too much to update the control policy frequently. Cognitive tasks suggest that, in such circumstances, using the inflexible habitual behavior carries a much smaller computational costs than the non-habitual one [53], [54].

### Which Brain Area could Control the Reoptimization Process?

Sensitivity to cost function [55] and switching from habitual to non-habitual behavior [32] point to the basal ganglia as a key area for the reoptimization of the control policy. Indeed, sensitivity to motor costs is one of the hallmarks of Parkinson Disease patients. These patients have trouble moving at fast speed [56], [57], despite having the ability to do so [57], which suggests that these patients are very sensitive to the energy cost or not sensitive to reward [14]. Interestingly, switching from habitual to non-habitual behavior also requires a recruitment of the frontal cortical-basal ganglia neural network [33], [54]. In the present experiment, reoptimization of the control policy might occur at the transition from the random to the blocked schedule or after a change in target width during the random schedule. In summary, both the trouble of PD patients with motor costs and neurophysiological studies of switching behavior suggest that the basal ganglia network is important for the reoptimization of the control policy.
