# Correction: Trial-to-Trial Reoptimization of Motor Behavior Due to Changes in Task Demands Is Limited

**DOI:** 10.1371/annotation/2aa90b9a-1634-4ab7-b5e3-a601b2326bb8

**Published:** 2013-06-17

**Authors:** Jean-Jacques Orban de Xivry

The author name is incorrectly provided in the citation. The following is the correct citation:

Orban de Xivry J-J (2013) Trial-to-Trial Reoptimization of Motor Behavior Due to Changes in Task Demands Is Limited. PLoS ONE 8(6): e66013. doi:10.1371/journal.pone.0066013 

